# A production strain of soybean nodule bacteria RZ300 Bradyrhizobium japonicum resistant to drying on the seed surface: cultural properties and genomic features

**DOI:** 10.18699/vjgb-26-47

**Published:** 2026-05

**Authors:** Yu.V. Kosulnikov, A.A. Kryukov, K.N. Berdysheva, A.I. Kovalchuk, A.P. Yurkov, Yu.V. Laktionov

**Affiliations:** All-Russian Research Institute for Agricultural Microbiology, Pushkin, St. Petersburg, Russia; All-Russian Research Institute for Agricultural Microbiology, Pushkin, St. Petersburg, Russia; All-Russian Research Institute for Agricultural Microbiology, Pushkin, St. Petersburg, Russia; All-Russian Research Institute for Agricultural Microbiology, Pushkin, St. Petersburg, Russia; All-Russian Research Institute for Agricultural Microbiology, Pushkin, St. Petersburg, Russia; All-Russian Research Institute for Agricultural Microbiology, Pushkin, St. Petersburg, Russia

**Keywords:** nodule bacteria, soy Glycine max L., Bradyrhizobium japonicum, microbial biologics, osmotic stress, genome-wide sequencing, osmotic stress resistance genes, клубеньковые бактерии, соя Glycine max L., Bradyrhizobium japonicum, микробные биопрепараты, осмотический стресс, полногеномное секвенирование, гены устойчивости к осмотическому стрессу

## Abstract

Pre-sowing treatment of cultivated legume seeds with nodule bacteria preparations is a standard agronomic practice. This is particularly important in soybean cultivation, as effective microsymbionts of soybeans are often absent from the soil. However, as many studies have shown, the efficacy of biopreparations depends largely on the survival of rhizobial cells on seeds during drying. In this study, we analyzed the viability of three production strains of Bradyrhizobium japonicum Kirchner (634b, 640 and RZ300) on soybean (Glycine max L.) seeds of various origins (varieties: EN Argenta, Bara and Prudence). The experiments evaluated several parameters: inoculant concentrations (10 and 100 %), drying temperatures (5, 15, and 25 °C), and protective polymer-carbohydrate formulations. The experiments revealed that the soybean variety had no noticeable effect on the viability of the studied rhizobial strains, while the strains themselves differed significantly in this regard. The RZ300 strain demonstrated the highest resistance to drying on soybean seeds. A comparative genomic analysis of this strain and the less resistant B. japonicum strain 634b revealed the presence of the opgC gene in the RZ300 strain (encodes the ОpgC protein involved in the biosynthesis of osmoregulated periplasmic glucans (OPGs)). This gene is absent in strain 634b and may potentially determine the increased resistance of nodule bacteria to drying on seeds. An evaluation of various protective formulations demonstrated that formulations based on 50 % sucrose provide the best protection, with rhizobia showing the highest resistance to drying at +5 °C. The results obtained in this study can be used both in the selection of effective inoculant strains and for providing technological support in the development of biological products. The genomic data support the development of genetic screening systems to identify promising strains and the potential introduction of the opgC gene into promising rhizobial strains to improve their manufacturability, i. e. to enable effective early seed inoculation.

## Abstract (ru)

Предпосевная обработка семян возделываемых бобовых культур препаратами клубеньковых бактерий является стандартной агрономической практикой, активно используемой при выращивании сои, эффективные микросимбионты которой часто отсутствуют в почве. В то же время, как показали многие исследования, эффект от применения биопрепарата во многом зависит от выживаемости ризобиальных клеток на семенах при высыхании. В настоящей работе проведен анализ жизнеспособности у трех производственных штаммов Bradyrhizobium japonicum, 643б, 640 и RZ300, на семенах сои Glycine max L. разного происхождения (сорта ЭН Аргента, Бара и Пруденс) в опытах, различающихся по разным параметрам: концентрациям инокулянта (10 и 100 %), температурам высушивания (5, 15, 25 °C) и составам защитных полимерно-углеводных композиций. В результате экспериментов обнаружено, что сорт сои не оказывал заметного влияния на жизнеспособность изучаемых штаммов ризобий, в то время как штаммы существенно различались по этому признаку. Наибольшую устойчивость к высыханию на семенах сои показал штамм RZ300. Сравнительный анализ генома этого штамма с геномом слабо устойчивого к высыханию штамма B. japonicum 634б позволил выявить наличие у штамма RZ300 гена opgC (кодирует белок ОpgC, участвующий в биосинтезе осморегулируемых периплазматических глюканов (OPGs)), который отсутствует у штамма 634б и, возможно, может определять повышенную устойчивость клубеньковых бактерий к высыханию на семенах. При изучении эффекта различных защитных композиций было отмечено, что лучшими защитными свойствами обладают составы на основе 50 % раствора сахарозы, а наибольшая устойчивость ризобий к высыханию проявляется при температуре +5 °C. Полученные в этой работе результаты могут быть использованы как в селекции эффективных штаммов- инокулянтов, так и в технологическом сопровождении при создании биопрепаратов. Данные, полученные при изучении геномов штаммов, представляют интерес как для разработки систем генетического скрининга при поиске перспективных штаммов, так и для изучения возможности введения генетических конструкций с геном opgC в перспективные штаммы ризобий для улучшения их технологичности, т. е. обеспечения возможности эффективной заблаговременной инокуляции семян.

## Introduction

An important agrobiological feature of soybeans is the ability to form a nitrogen-fixing legume-rhizobial symbiosis with nodule bacteria (Vavilov, Posypanov, 1983; Regar et al., 2017). At the same time, an effective symbiosis only occurs when active virulent soybean symbiont bacteria are present in the soil in sufficient quantities, which is rarely the case under field conditions and, thus, reduces the legume yield (Lamptey et al., 2014). To fully realize the potential of legume-rhizobial symbiosis, it is necessary to artificially introduce symbiotically effective nodule bacteria strains into the rhizosphere, which in agricultural practice is achieved through the pre-sowing inoculation of soybean seeds with rhizobialbased biopreparations. A biopreparation based on the Bradyrhizobium japonicum 634b strain under the trade name Rhizotorphin is widely used in domestic agriculture and provides high yield increases across various soybean varieties (Vasilchikov, Gurev, 2018; Volobueva et al., 2023). Strain 634b is frequently used as a reference for assessing new rhizobial candidates, including strain 640 (Magomedov et al., 2011).

When analyzing the effectiveness of rhizobial strains, seed inoculation is carried out on the day of sowing, as it is known that nodule bacteria are sensitive to drying. However, in agricultural practice, for both technological and economic reasons, pre-sowing seed treatment is carried out well in advance, which can lead to the death of the applied rhizobia even before the seeds are planted. A review of this problem was provided by J. Vriezen et al. (2007). As early as 1932, E.B. Fred et al. reported a decrease in the number of viable nodule bacteria cells on seeds and suggested that the nutrient medium composition, pH and temperature are factors determining the resistance of cells to desiccation

Later, J.M. Vincent et al. (1961) studied the culture of Rhizobium trifolii during its drying on glass beads and proposed that the decrease in the number of viable rhizobia was due to both “seed factors” and the drying factor itself. It was shown that the negative effect of drying could be partially offset by the addition of saccharides, particularly maltose, which indicates that the availability of nutrients, and potentially other dissolved substances, affects the survival of bacterial cells

Several studies have shown the difference in the survival dynamics for nodule bacteria on various matrices such as glass beads, seeds, soil, nitrocellulose filters, etc. (Vriezen et al., 2006). One possible reason for the observed differences is that dry inoculated seeds have a water activity ranging from 0.45 to 0.6 (Smith, 1992) and, thus, still contain a relatively large amount of water compared with completely dry surfaces of glass beads or nitrocellulose filters.

From a practical point of view, given the high sensitivity of nodule bacteria to drying, it is important either to sow the treated seeds on the day of treatment (Vasilchikov, Gurev, 2018), or to use special polymer and carbohydrate protectors that increase the resistance of bacteria to osmotic stress (Skorupska et al., 2006; Deaker et al., 2007; Reina-Bueno et al., 2012). In particular, we have previously shown that the watersoluble polymer polyvinylpyrrolidone combined with activated carbon significantly improves rhizobial survi- val on inoculated seeds. This combination is more effective that polyvinylpyrrolidone alone and it reduces bacterial mortality on inoculated seeds by 20–30 % after the first 5–7 days of seed storage (Laktionov et al., 2019).

Finally, it should be noted that studies on the survival of rhizobia during pre-sowing inoculation should become an important part in the selection of effective inoculant strains. A key element of such research should be the investigation of the mechanisms of rhizobia resistance to desiccation. While a number of studies have explored the molecular and genetic aspects of osmotic resistance in nodule bacteria (Vriezen et al., 2007), especially in the context of climate change (Zhang et al., 2024), relatively few studies have evaluated the viability of rhizobial collection strains on legume seeds or explored methods to enhance the resistance of cells to osmotic stress (Laktionov et al., 2019).

The aim of this study was to evaluate the resistance of three production strains of soybean nodule bacteria: strain 634b (used to produce inoculants for Risotorphin), strain 640 (identified in several studies as more effective than strain 634b) and a new promising strain RZ300. The evaluation was conducted on various soybean varieties under different storage temperatures and using protective polymer-carbon formulations of varying compositions. Furthermore, the study aimed to elucidate the mechanisms underlying the desiccation resistance of these rhizobial strains using comparative genomics.

## Materials and methods

The study used B. japonicum strains 634b, 640 and RZ300 from the network bioresource collection in the field of genetic technologies for agriculture (Russian Collection of Agricultural Microorganisms RCAM).

Strain 634b was isolated from four soybean (cv. Kolkhida 4) nodules at the Natshakai Station in Georgia. This strain is used to produce inoculants for soy under the trademark Risotorphin (Vasilchikov, Gurev, 2018; Volobueva et al., 2023). Strain 640 was isolated in 1976 from soybean (cv. Smena) nodules in the meadowchernozem soil of the Amur region at the All-Russian Soybean Research Institute. A number of studies have identified the strain as more effective than 634b (Magomedov et al., 2011). The RZ300 strain was isolated in 2022 in the Krasnodar Territory from soybean (cv. Bara) nodules and has hardly been studied to date. Strain RZ300 exhibits stable nodulation in both chamber and field experiments under normal and extreme conditions (including presence of chemical pesticides, and lack of moisture). Patent No. 2806593 “Method of cultivation of nodule bacteria of soy Bradyrhizobium japonicum RZ300” was obtained for strain RZ300.

B. japonicum strains 634b, 640 and RZ300 were grown for inoculation in a liquid semi-synthetic medium (Yadav et al., 2011): Mannit – 10.0 g/L; yeast extract – 1 g/L; K2HPO4 – 0.5 g/L; MgSO4 · 7H2O – 0.2 g/L; NaCl – 0.1 g/L; CaCO3 – traces. Cultures were grown for 7 days at 28 °C in 250 mL glass flasks with cotton stoppers on an orbital shaker at 180 rpm. After cultivation, the flasks were stored at +5 °C. To obtain experimental liquid cultures, a 2 % (v/v) inoculum from the flasks was aseptically transferred to a BIORUS laboratory fermenter using the same medium composition (Yadav et al., 2011).

The microorganisms were cultured to the stationary phase, in which the cells are most resistant to osmotic stress (Soria et al., 2006), in a periodic regime for 7 days at 28 °C with mechanical mixing (150 rpm) and aeration (1 L air/1 L medium per min). The resulting experimental preparations were aseptically poured into sterilized glass flasks with cotton stoppers and stored in a refrigerator. To determine the titer of the resulting bacterial suspensions, a series of consecutive 10-fold dilutions were prepared, followed by inoculation on Petri dishes with an agar medium (Yadav et al., 2011); the number of colonies was counted after 10 days of incubation at 28 °C.

To study the resistance of strains to drying, glass beads and Glycine max L. soybean seeds from three varieties of different origin – EN Argenta (Deriglazova, Morozov, 2022), Bara (Parakhin et al., 2017), Prudence (Bobkova, 2020) – were used.

The dynamics of nodule bacteria viability during drying were monitored for 24 hours following inoculation with rhizobium preparations. To determine the effect of the “seed factor”, glass beads of a similar size to the seeds were used. The preparations were applied in concentrations of 10 % (aqueous solution) and 100 % (undiluted inoculant) at the rate of 10 L per 1 ton of seeds, following standard agricultural practice (Kincharova, Matvienko, 2021). An undiluted preparation was applied to assess the effect of exopolysaccharides and residual media, as well as a high cell density. The treatment of seeds and beads with tank solutions followed by an assessment of the dynamics of bacterial viability over time was carried out according to the author’s methodology (Laktionov et al., 2019). Experiments were conducted in triplicate.

To evaluate the effectiveness of polymer-carbohydrate compositions as bacterial protectors on seeds drying at various temperatures, the following formulations were prepared: 1 % solution of carbokimethylcellulose (CMC); 50 % solution of sucrose; a mixture of 1 % solution of carbokimethylcellulose (CMC), 50 % solution of sucrose; a mixture of 1 % solution of carbokimethylcellulose (CMC), 50 % solution of sucrose and 1 % activated carbon.

The purity of microbial cultures was determined by both morphological characteristics (morphology of colonies on plates and rhizobium cells in a fixed smear) and molecular genetic identification (16S rRNA gene sequence analysis).

Rhizobium DNA was isolated by the CTAB method (Doyle J.J., Doyle J.L., 1987, 1990). Whole-genome sequencing for strains RZ300 (BioProject PRJNA1266151) and 634b (BioProject PRJNA1334995) was performed using 3rd generation sequencing (Oxford Nanopore Technologies, UK). Genome assembly and annotation were conducted using bioinformatic tools including: flye, SPAdes, mauveAligner, GeneMark, Prokka, and EggNOG-mapper. Annotated whole-genomic sequences were deposited GenBank.

Statistical analysis was performed using the SciPy library, and data visualization was performed using the matplotlib library in Python.

## Results

At the first stage of the research, the cell titers of the bacterial suspensions were determined at the time of the working solution preparation. The titers were: 1.8 ± 0.24 · 109 CFU/mL for strain 634b; 1.77 ± 0.29 · 109 CFU/mL for strain 640; 2.0 ± 0.19 · 109 CFU/mL for strain RZ300.

**Fig. 1.**
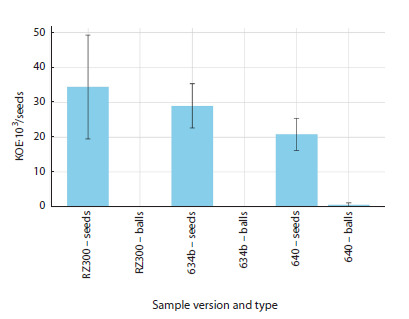
Cell viability of B. japonicum strains on EN Argenta soybean seeds and glass beads 24 hours after inoculation.

To evaluate the specific effects of drying on the survival of the studied strains, rhizobial viability on soybean seeds (cv. EN Argenta) and glass beads was compared (the results are shown in Figure 1). It has been shown that viable cells on glass beads remained at levels of several thousand CFU per bead only for three hours after inoculation and almost completely died within a day after treatment. In contrast, tens of thousands of viable cells remained on the seeds a day after treatment. At the same time, the differences in cell viability between different strains were not statistically significant, the calculated F-statistic (2.67) was less than the critical F-value (3.89) at a 5 % significance level.

**Fig. 2.**
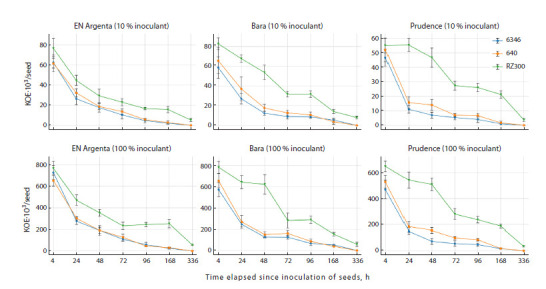
Survival dynamics of B. japonicum strains on soybean seeds (cv. EN Argenta, Bara, Prudence), inoculated with 10 and 100 % solutions of preparations without the addition of protective formulations.

In the second stage of the research, the potential influence of three soybean varieties on the dynamics of nodule bacteria viability was studied without the addition of protective formulations (Fig. 2). When seeds were treated with a 10 % inoculant solution at a rate of 10 L/t, 168 hours post-inoculation, the cells of the RZ300 strain remained on soybean seeds of the EN Argenta, Bara and Prudence varieties in the amount of 16, 14 and 21 thousand CFU per seed, respectively. At the same time, for strains 634b and 640, these values did not exceed 3, 6 and 2 thousand CFU per 1 seed, respectively.

Viability analysis of the three B. japonicum strains across different soybean varieties 168 hours after treatment showed that for the EN Argenta variety, the number of viable cells of the RZ300 strain (X = 15.67 thousand CFU/seed) was significantly higher (LSD = 5.19, p < 0.05) than that of strains 634b (X = 2.00 thousand CFU/seed) and 640 (X = 2.67 thousand CFU/seed). Similar patterns were observed for the Bara and Prudence varieties. The RZ300 strain demonstrated statistically higher cell viability in the Bara (X = 14.00 thousand CFU/seed; LSD = 4.85) and Prudence (X = 21.33 thousand CFU/seed; LSD = 3.20) varieties compared to strains 634b and 640, between which no significant differences were found. After 336 hours, the number of bacteria of the RZ300 strain on seeds of all varieties was estimated at thousands of CFU/seed, while viable cells of strains 634b and 640 could not be isolated from seeds. A similar pattern was observed for the 100 % inoculant solutions, accounting for the higher initial cell densities.

**Fig. 3.**
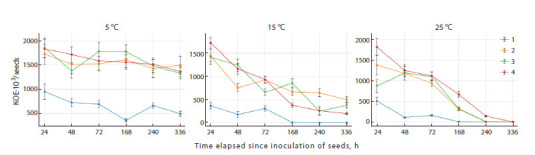
Dynamics of the viability of RZ300 cells on soybean seeds of the EN Argenta variety during two weeks of storage at different temperatures (+5, +15, +25 °C) and with various protective formulations (1 – 1 % CMC; 2 – 50 % sucrose, 3 – 1 % CMC + 50 % sucrose; 4 – 1 % CMC + 50 % sucrose + 0.5 % activated carbon).

In the third stage of the study, the effect of various protective formulations and temperature conditions on cell viability of the RZ300 strain, which demonstrated the best survival rates in inoculated seeds, was studied. Figure 3 shows that the rate of cell mortality increased with increasing temperature. At the same time, carboxymethylcellulose was a relatively ineffective cell protector, while sucrose and sucrose-based compositions significantly increased the resistance of bacteria to drying on seeds. At +5 °C, the temperature exhibiting the least contrast between treatment means, it was shown that the sucrose variants provided significantly higher cell viability (at 5 % significance level) compared with 1 % carboxymethylcellulose, while the differences among various sucrose-based formulations were not significant. Thus, it can be assumed that sucrose has the greatest protective effect for bacteria undergoing drying on seeds. Consequently, sucrose-based compositions can further enhance the resistance of the RZ300 strain to drying on seeds.

Finally, to identify the genomic features that may be responsible for resistance of strain RZ300 to desiccation, a comparative analysis of its whole genome was performed against the genome of the less resistant strain 634b.

The results of whole-genome sequencing for the RZ300 strain have been deposited in NCBI (BioProject PRJNA1266151). The size of the circular chromosome is 9,199,961 bp. The total number of genes is 9,643, of which there are 5,736 coding ones, and a significant part is identified as pseudogenes – 3,842. The results of whole-genome sequencing for strain 634b have also been deposited in NCBI (BioProject PRJNA1334995). The genomes of the RZ300 and 634b strains were compared using the RAST (Rapid Annotation using Subsystem Technology) server. The Table shows the differentially represented genes specific to each studied strain. The genes putatively associated with the increased stress resistance of the RZ300 strain are highlighted in bold.

**Table 1.**
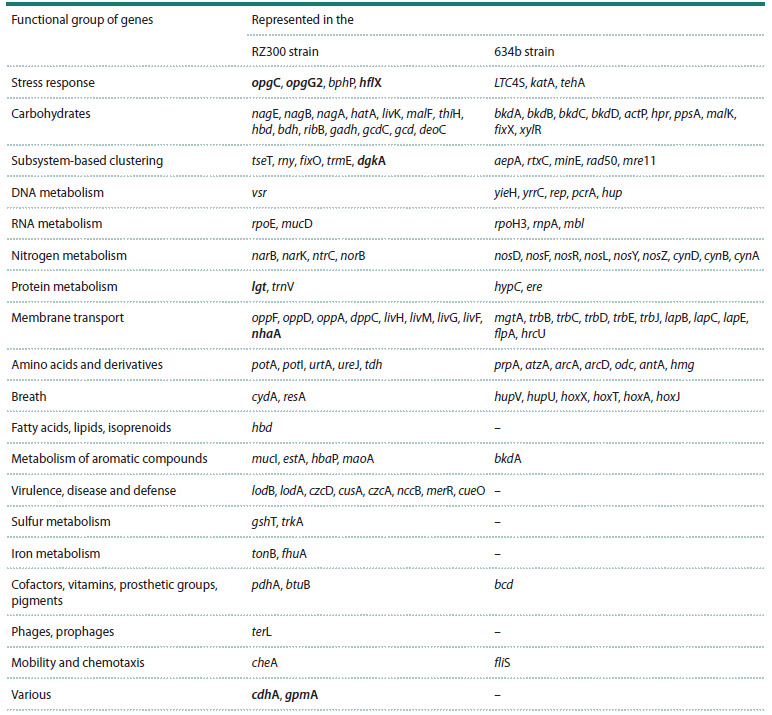
Comparative analysis of functional genes groups in drying-resistant RZ300 and drying-sensitive 634b of B. japonicum strains Note. Genes presumably related to the increased stress resistance of strain RZ300 are highlighted in bold.

## Discussion

In agricultural practice, the productivity potential of legume-rhizobial symbiosis can be realized only if high bacterial survival is maintained on the seeds prior to sowing. At the same time, despite the high practical importance of the issue of nodule bacteria resistance to drying on seeds, this area of research remains poorly studied in both Russian and international scientific literature.

The use of glass beads has shown that the surface of seeds is a more favorable environment for nodule bacteria than the inert surface of glass. This indicates that the evolutionary history of symbiosis, in addition to the well-known functions related to virulence and nitrogen fixation, may have included the development of specific functions related to maintaining the viability of rhizobia on the seed surface, which is obviously of great importance for ensuring the stability of symbiosis.

Notably, our findings indicate that the seed variety does not significantly affect the dynamics of the decrease in the number of cells, whereas bacterial strains differ significantly in their resistance to desiccation. This highlights two points: first, the conservative nature of the plant-side mechanisms that ensure the preservation of rhizobia on the surface of seeds, and second, the potential for selecting inoculant strains based on resistance to desiccation as a way to increase their manufacturability, that is, integration with existing agrotechnological schemes, which has received very little attention so far.

In this study, we have shown that the selection of effective protective formulations and seed treatment methods significantly contributes to ensuring the survival of rhizobia on inoculated seeds. Thus, for instance, a 10-fold increase in the consumption rate of the inoculant led to a proportional increase in the number of viable cells on the seeds throughout the study period. However, the overall dynamics of cell death remained similar to that when using 10 % solutions. This suggests that there is no pronounced protective effect of exopolysaccharides applied with an increased volume of the inoculant on the osmotic resistance of the strains.

It has been shown that the dynamics of reducing the number of viable bacteria on seeds slows down significantly with decreasing temperature, as well as with the addition of protective polymer-carbohydrate compositions, in particular, based on 50 % sucrose. Thus, the combination of lowering the temperature and adding sucrose during the processing of soybean seeds can ensure a significantly higher number of viable cells per seed at the time of planting.

Comparative analysis of the genomes of strain RZ300 and strain 634b revealed the following differences. According to the group of “stress response” genes, the drying-resistant strain RZ300 has the opgC and opgG2 genes responsible for the synthesis of osmoregulated periplasmic glucans, which directly explains its osmotic stress tolerance, while the osmosensitive 634b lacks these genes. OpgC is a protein involved in the biosynthesis of osmoregulated periplasmic glucans (OPGs), occurring in a number of bacteria. These glucans play an im- portant role in adaptation to osmotic stress, cell envelope integrity, biofilm formation, and pathogenicity. It is likely that the role of this gene in the formation of biofilms determines the resistance of bacteria to desiccation on seeds. Mutations in the opgC gene can lead to changes in OpgC synthesis, which, in turn, affects bacterial survival under osmotic stress (Bontemps-Gall, Lacroix, 2015).

Among other genes that could potentially be involved in the control of stress resistance, the RZ300 strain contains a gene encoding a GTP-binding protein related to HflX. The results of recent studies indicate the role of HflX in determining bacterial resistance to macrolides and lincosamides (Rudra et al., 2020). Another important candidate gene for stress control represented in strain RZ300 is dgkA. It is known that the expression of the dgkA gene is associated with accelerated bacterial growth and survival in response to adverse environmental factors (Baker et al., 2021). In the context of membrane transport, the drying-resistant strain RZ300 demonstrates more advanced import systems, including transporters for oligopeptides (oppA, oppD, oppF genes), dipeptides (DDPPC genes) and amino acids (livH, livM, livG, livF), and also has the nhaA gene for regulating Na+/H+- antiport. It is noteworthy that the regulation of the HAA gene of Escherichia coli is used to produce transgenic rice plants (Oryza sativa L. ssp. japonica) with increased resistance to salinity, but reduced one to drought (Wu et al., 2005).

A notable feature of the RZ300 is the presence of the lgt gene encoding an enzyme involved in lipoprotein biosynthesis, which is critically important for maintaining the integrity of the cell wall and, consequently, for resistance to stress factors. It has been shown that a decrease in Lgt levels in a clinical strain of uropathogenic E. coli leads to increased permeability of the outer membrane and increased sensitivity to serum and antibiotics (Diao et al., 2021). Finally, the cdhA gene encoding choline dehydrogenase and the gpmA gene responsible for phosphoglycerate mutase were found in the RZ300 strain, which indicate its ability to maintain the integrity of cell membranes.

A number of studies have shown that pathogenic bacteria mutated by the cdhA gene have reduced virulence and resistance to antibiotics acting on the cell wall (Pancholi et al., 2010). It is known that a deletion in the gpmA gene in E. coli leads to a specific hypersensitivity to H2O2, comparable to the deletion of the main H2O2 scavenger gene katG. Exposure to H2O2enhances the transcription of gpmA, which highlights its role in protecting against oxidative stress. Thus, the gpmA gene can be defined as an element of the bacterial defense mechanism against oxidative stress (Roth et al., 2022).

Thus, the analysis of the genetic differences between the osmosis-resistant strain RZ300 and the osmosensitive 634b identified a large group of differentially represented genes, putatively associated with resistance to drying control. Among these, opgC and opgG2 are of the greatest interest, responsible for the synthesis of osmoregulated periplasmic glucans, which can play an important role in determining the resistance of nodule bacteria to desiccation on inoculated seeds. These data not only clarify the mechanisms of rhizobia resistance to desiccation, but also provide future research directions such as transferring of genetic constructs containing these genes into symbiotically effective rhizobia strains to meet the requirements of modern agricultural technologies.

## Conclusion

In this study, the problem of ensuring the resistance of rhizobial inoculant strains to drying on the surface of inoculated soybean seeds is investigated in detail. We demonstrated that the survival rate of rhizobia strains on soybean seeds, firstly, is significantly higher than on a neutral carrier (glass beads), and secondly, does not depend on the soybean variety. It has been shown that the main differences in survival are related to the strains themselves, which indicates the importance of selecting promising inoculant strains based on this feature. Optimal technologies have been developed for the use of protective compositions that increase the resistance of strains to drying on seeds. To clarify the underlying mechanisms of strain resistance to desiccation, a comparative whole-genome analysis was performed on resistant and less resistant strains. Candidate genes potentially involved in the control of this trait were identified.

Of particular interest is the presence of the opgC gene in the resistant RZ300 strain, which is absent in the less resistant 634b strain and likely plays a role in bacterial resistance to desiccation on seeds. We assumed that this gene may serve as a molecular marker of resistance to desiccation of bacteria on seeds and can be used in the future for targeted breeding or genetic modification of rhizobial strains for microbial biopreparations.

## Conflict of interest

The authors declare no conflict of interest.
